# CRF Induces Intestinal Epithelial Barrier Injury via the Release of Mast Cell Proteases and TNF-α

**DOI:** 10.1371/journal.pone.0039935

**Published:** 2012-06-29

**Authors:** Elizabeth L. Overman, Jean E. Rivier, Adam J. Moeser

**Affiliations:** 1 College of Veterinary Medicine, North Carolina State University, Raleigh, North Carolina, United States of America; 2 The Salk Institute, La Jolla, California, United States of America; Duke University Medical Center, United States of America

## Abstract

**Background and Aims:**

Psychological stress is a predisposing factor in the onset and exacerbation of important gastrointestinal diseases including irritable bowel syndrome (IBS) and the inflammatory bowel diseases (IBD). The pathophysiology of stress-induced intestinal disturbances is known to be mediated by corticotropin releasing factor (CRF) but the precise signaling pathways remain poorly understood. Utilizing a porcine *ex vivo* intestinal model, the aim of this study was to investigate the mechanisms by which CRF mediates intestinal epithelial barrier disturbances.

**Methodology:**

Ileum was harvested from 6–8 week-old pigs, mounted on Ussing Chambers, and exposed to CRF in the presence or absence of various pharmacologic inhibitors of CRF-mediated signaling pathways. Mucosal-to-serosal flux of 4 kDa-FITC dextran (FD4) and transepithelial electrical resistance (TER) were recorded as indices of intestinal epithelial barrier function.

**Results:**

Exposure of porcine ileum to 0.05–0.5 µM CRF increased (p<0.05) paracellular flux compared with vehicle controls. CRF treatment had no deleterious effects on ileal TER. The effects of CRF on FD4 flux were inhibited with pre-treatment of tissue with the non-selective CRF_1/2_ receptor antagonist Astressin B and the mast cell stabilizer sodium cromolyn (10^−4 ^M). Furthermore, anti-TNF-α neutralizing antibody (p<0.01), protease inhibitors (p<0.01) and the neural blocker tetrodotoxin (TTX) inhibited CRF-mediated intestinal barrier dysfunction.

**Conclusion:**

These data demonstrate that CRF triggers increases in intestinal paracellular permeability via mast cell dependent release of TNF-α and proteases. Furthermore, CRF-mast cell signaling pathways and increases in intestinal permeability require critical input from the enteric nervous system. Therefore, blocking the deleterious effects of CRF may address the enteric signaling of mast cell degranulation, TNFα release, and protease secretion, hallmarks of IBS and IBD.

## Introduction

The gastrointestinal barrier, consisting primarily of intestinal epithelial cells, mucus layer, and sub-epithelial immune cells, selectively controls the access of the immense luminal load of antigens and resident microorganism to the underlying lamina propria immune tissues [1]. It is well-known that intestinal barrier function can be adversely affected by acute or chronic psychological stress, resulting in increased intestinal permeability [2], [3], [4], [5], [6], a critical event in the onset of clinical symptoms of GI disorders including irritable bowel syndrome (IBS) and inflammatory bowel disease (IBD) [7], [8], [9], [10]. Disturbances in intestinal barrier facilitates bacterial movement from the lumen into the lamina propria while also critically impairing other vital functions including absorption of nutrients, transport of ions, secretion [11], [12], motility, and visceral hypersensitivity [13], [14]. Although it is known that stress compromises intestinal barrier function, the precise mechanisms remain poorly understood.

CRF is a 41 amino acid peptide, produced in the central nervous system and peripheral tissues [15], [16] in response to stress and has been shown to play a central role in stress-induced intestinal pathophysiology. CRF activity is mediated by activation of specific seven transmembrane G-protein coupled receptors (GPCRs) known as CRF_1_ and CRF_2._ CRF has been shown to induce intestinal barrier disturbances in multiple animals and human tissues models. Several studies have demonstrated that CRF mediates its effects via mast cell activation [6], [11], [17]. Upon activation, mast cells are capable of releasing a variety of pro-inflammatory mediators, including de novo synthesized mediators such as prostaglandins, leukotrienes, and cytokines or preformed granule-housed mediators including histamine, serine proteases, tryptase, chymase, and cytokines [18], which profoundly influence intestinal epithelial barrier function; however, the mast cell mediators and signaling pathways that are responsible for CRF-mediated intestinal barrier dysfunction have not been fully elucidated. Here, utilizing a porcine model, we investigated the mechanisms of CRF-mediated intestinal epithelial barrier dysfunction.

## Results

### Influence of CRF on Porcine Ileal Intestinal Barrier Function

**Figure 1:**
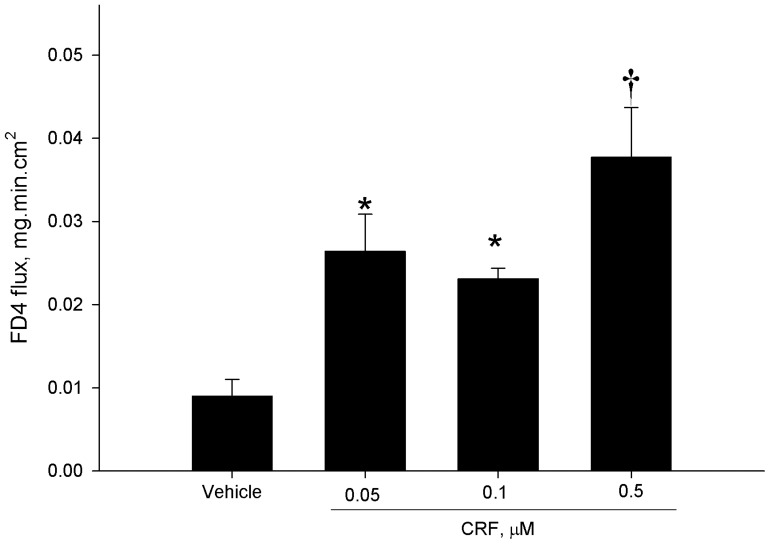
CRF induces elevations in FD4 flux in porcine ileum. Porcine ileum was placed on Ussing Chambers and treated with CRF at increasing concentrations (0.05, 0.1, and 0.5 µM) and the rate of FD4 flux was measured over a 180-minute period. CRF at all concentrations induced elevations in FD4 flux (p<0.01) with the highest FD4 flux rates observed with 0.5 µM CRF. Data for each experimental treatment are expressed as means ± SE for n = 6−8 pigs. Symbols (*,†) differ from other treatments by p<0.05; ANOVA.

**Figure 2:**
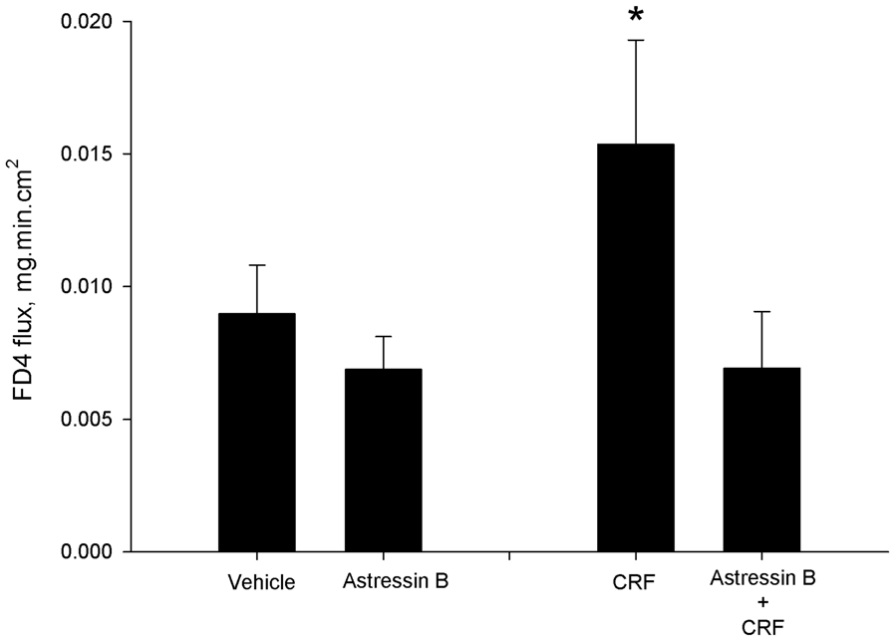
CRF-induced FD4 flux is Prevented with Astressin B. CRF (0.5 µM) induced elevations in the rate of FD4 flux across the porcine ileum mounted on Ussing chambers. Pre-treatment of ileal mucosa with Astressin B (1 µM) 30 minutes prior to CRF exposure prevented CRF-mediated increases in FD4 flux rates. Data for each experimental treatment are expressed as means ± SE for n = 6−8 pigs. Symbols (*,†) differ from other treatments by p<0.05; ANOVA.

**Figure 3:**
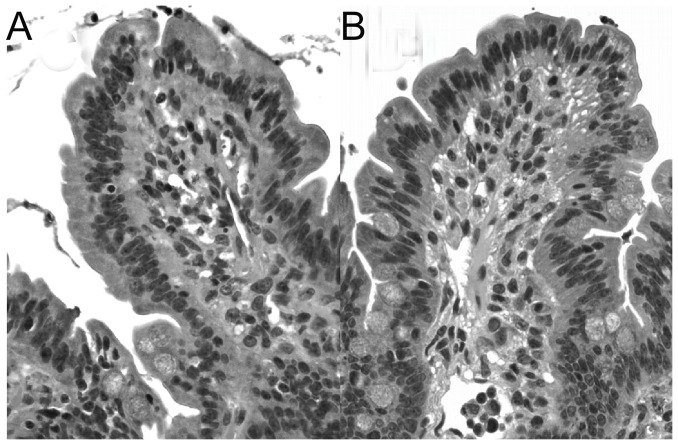
Histological appearance of CRF-treated porcine ileal mucosa on Ussing chambers. Ileal histology (20× magnification) of hematoxylin and eosin-stained porcine ileal sections after 180 minutes on Ussing chamber following treatment with vehicle control (A) or 0.5 µM CRF (B). Images are representative of ileal sections from n = 6 pigs/treatment.

**Figure 4:**
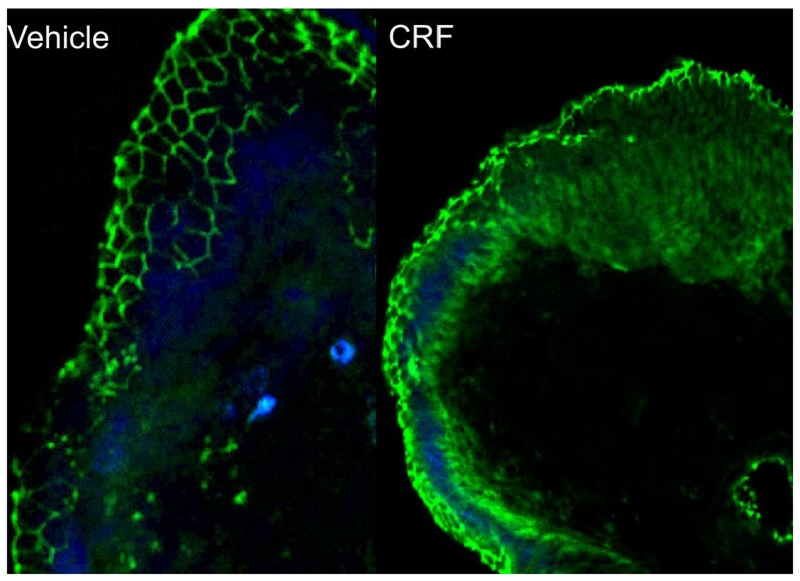
Occludin localization in porcine ileum exposed to CRF. In control (vehicle-treated) tissues (A), occludin (green) was localized predominantly to the interepithelial tight junctions as demonstrated by the epithelial membrane staining patterns whereas CRF-treated tissues (B) exhibited a disrupted occluding staining pattern. Images are representative of tissues from n = 3 pigs.

We employed an *ex vivo* Ussing chamber system to investigate the role of local CRF signaling on intestinal epithelial barrier function in the porcine ileum. CRF, at concentrations of 0.05 and 0.1 µM, and 0.5 µM induced elevations in FD4 flux across ileal mucosa compared with vehicle-treated controls (Figure 1). In contrast, exposure of ileal mucosa to CRF did not influence TER over the 180 minute time period on the chambers (data not shown). To confirm that CRF was mediating its effects on intestinal permeability via CRF receptors, ileal mucosa was pre-treated with the CRF receptor antagonist, Astressin B (1 µM), prior to exposure of CRF (0.5 µM). Astressin B prevented CRF-induced elevations in FD4 flux (Figure 2). Histological analysis revealed no disruption of intestinal epithelial continuity with CRF (0.5 µM) treatment (Figure 3) indicating that the effects of CRF were due to alterations in the paracellular flux pathways rather than destruction of the epithelium. Immunoflourescence analysis of the tight junction protein occludin revealed marked disruption in occludin staining patterns in ileal tissues exposed to CRF (Figure 4).

### Mast Cell Degranulation is required for CRF-mediated Increases in Paracellular Permeability in the Porcine Ileum

**Figure 5:**
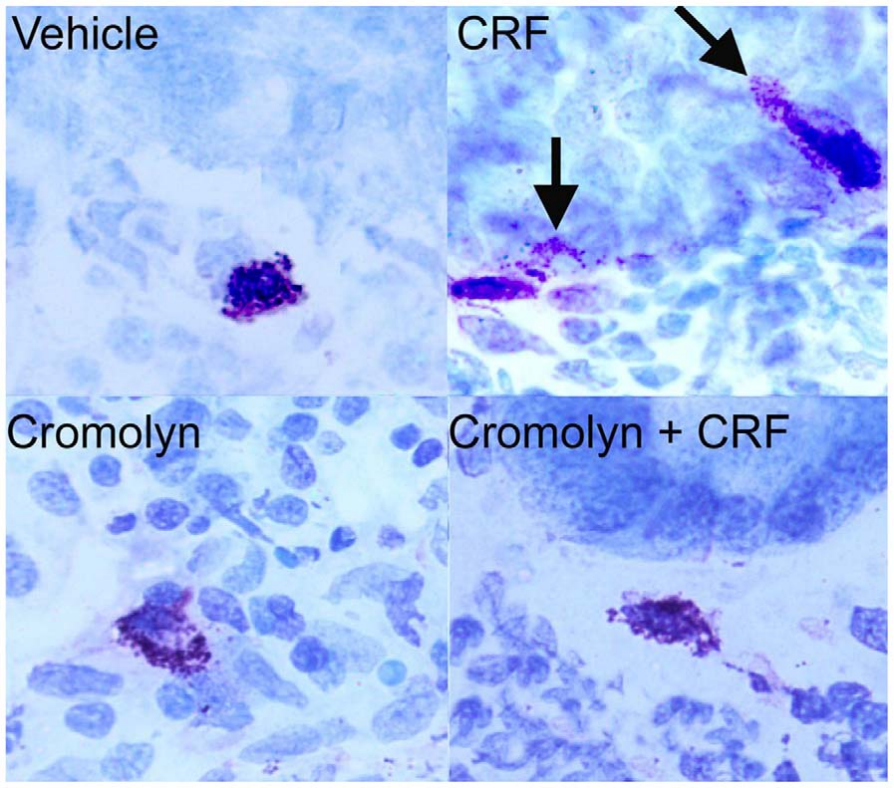
Histologic analysis of CRF-mediated intestinal mast cell degranulation. Treatment of porcine ileal tissues mounted on Ussing chambers with CRF caused and increase in mast cell degranulation as determined by toluidine blue staining. Arrows indicate the release of mast cell granules into surrounding tissues. Sodium cromolyn, a mast cell stabilizing agent, prevented CRF-induced mast cell degranulation. Figures are representative of ileal tissue sections from n = 4 pigs.

**Figure 6:**
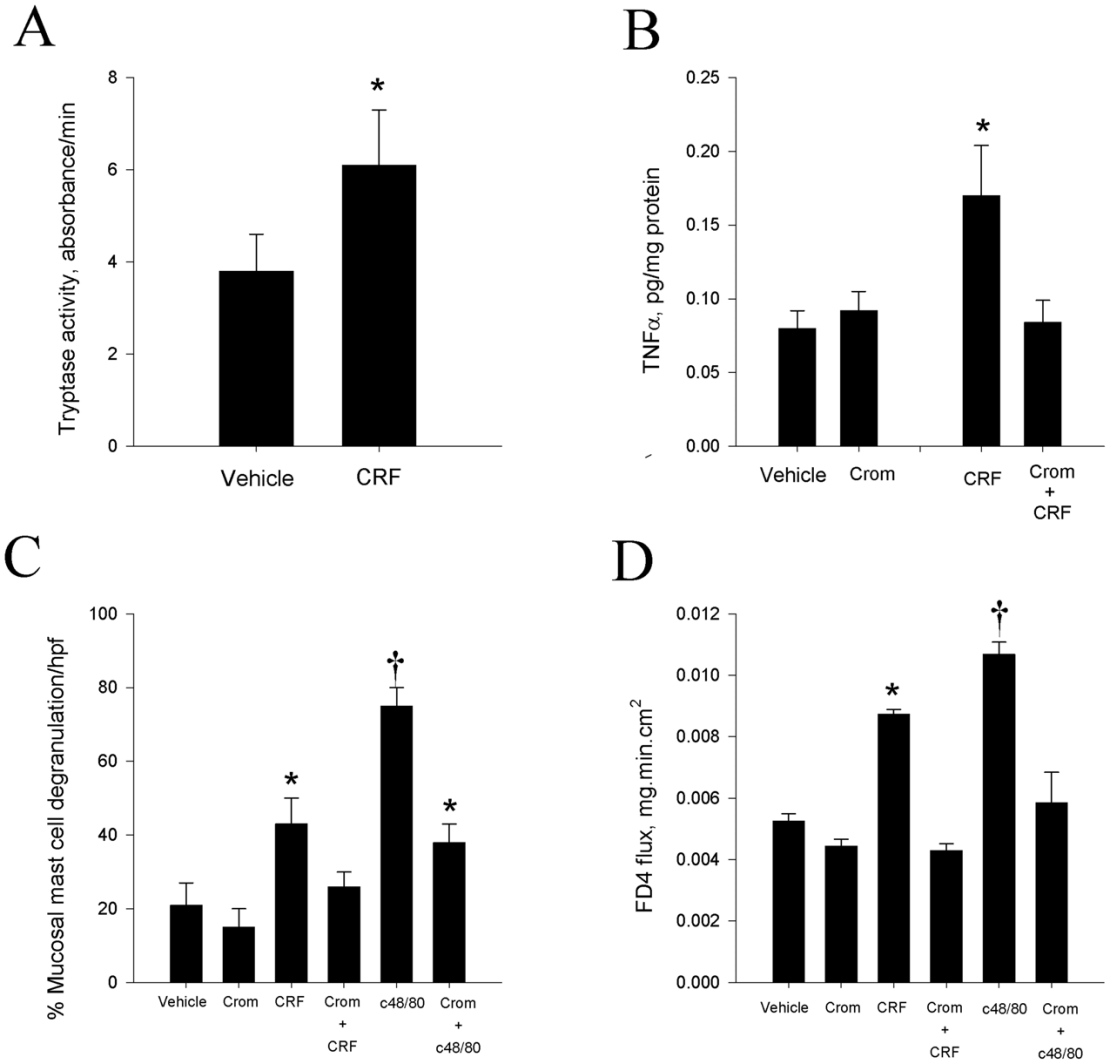
The role of mast cell activation in CRF-mediated changes in FD4 flux. Treatment of porcine ileal tissues mounted on Ussing chambers with CRF increased mast cell degranulation indicated by the increased percentage of degranulated mast cells (C) and the increased release of mast cell tryptase (A) and TNF-α (B). Treatment of porcine ileal tissues with cromolyn sodium 30 minutes prior to exposure to 0.5 µM CRF blocked mast cell degranulation (C) and TNF-α release (B) and prevented CRF-mediated increases in FD4 permeability (D). c48/80 (5 µg/mL) induced marked degranulation of mast cells (C) and increases in FD4 flux (D) that were inhibited with sodium cromolyn. Data for each FD4 flux experiments are expressed as means ± SE for n = 6−8 pigs. Symbols (*,†) differ from other treatments by p<0.05; ANOVA.

To determine the role of mast cell activation in CRF-mediated intestinal permeability, we evaluated determined if CRF triggered mast cell activation in the porcine ileum and whether blockade of mast cell activity alterned CRF-mediated disturbances in intestinal epithelial permeability. Exposure of porcine ileum to 0.5 µM CRF induced mucosal mast cell degranulation as determined by histological evidence of mast cell degranulation (Figure 5) and quantified by the increased percentage of degranulated of intestinal mucosal mast cells (Figure 6C). Furthermore, CRF induced the release of mast cell tryptase (Figure 6A) and TNFα (Figure 6B). Pre-treatment of porcine ileal tissues with the mast cell stabilizing agent, sodium cromolyn (10^−4^ M), blocked CRF-mediated mast cell degranulation (Figures 5, 6C) and increases in FD4 flux (Figure 6D). Cromolyn also inhibited elevations in TNFα (Figure 6D) thus indicating that CRF-induced TNFα release was mast cell dependent. To compare the CRF response to a known mast cell degranulation response, tissues were treated with the mast cell degranulating compound c48/80. c48/80 induced marked mast cell degranulation (Figure 6C) and increases in FD4 flux (Figure 6D) that were greater than CRF (p<0.05).

### Blockade of TNF-α and Protease Activity Prevents CRF-induced Gut Barrier Dysfunction

**Figure 7:**
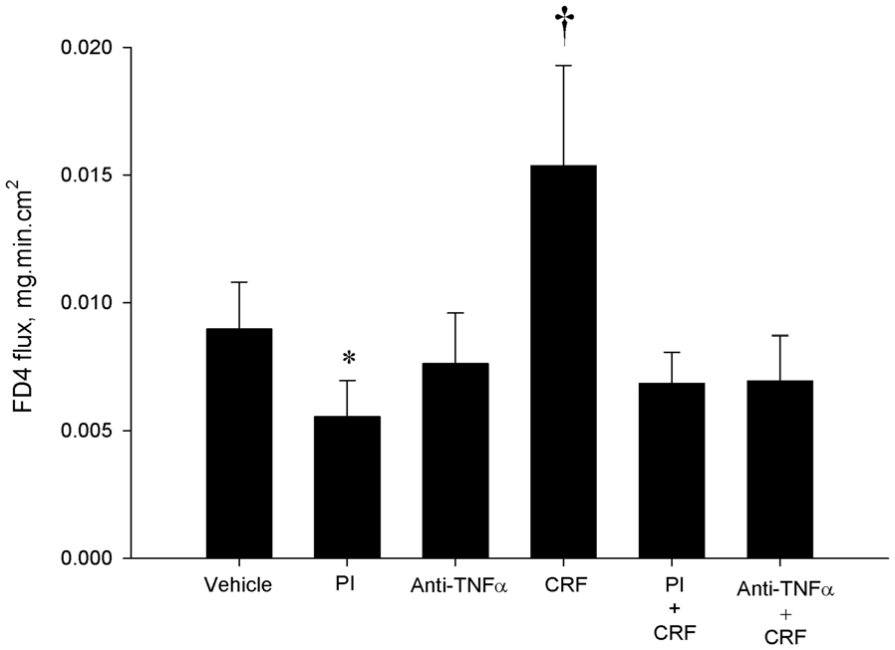
Neutralization of TNF-α and protease activity inhibits CRF-induced increases in FD4 flux in porcine ileum. Pretreatment of porcine ileum mounted on Ussing chambers with neutralizing anti-TNF-α antibody prevented CRF –induced increased in FD4 flux. Pre-treatment of porcine ileum with a protease inhibitor (PI) cocktail reduced baseline FD4 flux values and CRF-induced increases in FD4 flux. Values represent means SE; *n = *6−8 animals. Symbols (*,†) differ from other treatments by p<0.05.

To determine if the mast cell mediators, TNF-α and MC proteases, were responsible for CRF-mediated intestinal barrier dysfunction in the porcine ileum, we treated porcine ileal mucosa on Ussing chambers with a PI cocktail or porcine anti-TNF-α prior to CRF exposure. Protease inhibitors (PI) and anti-TNF-α (0.1 and 10 µg/mL) inhibited CRF-stimulated elevations in FD4 flux (Figure 7A–B). Under baseline condition (no CRF), PI and the higher anti-TNF-α (10 µg/mL) reduced baseline FD4 flux indicating the influence of proteases and TNF-α under basal conditions.

### Neuronal Activity is required for CRF-induced Gut Barrier Dysfunction

**Figure 8:**
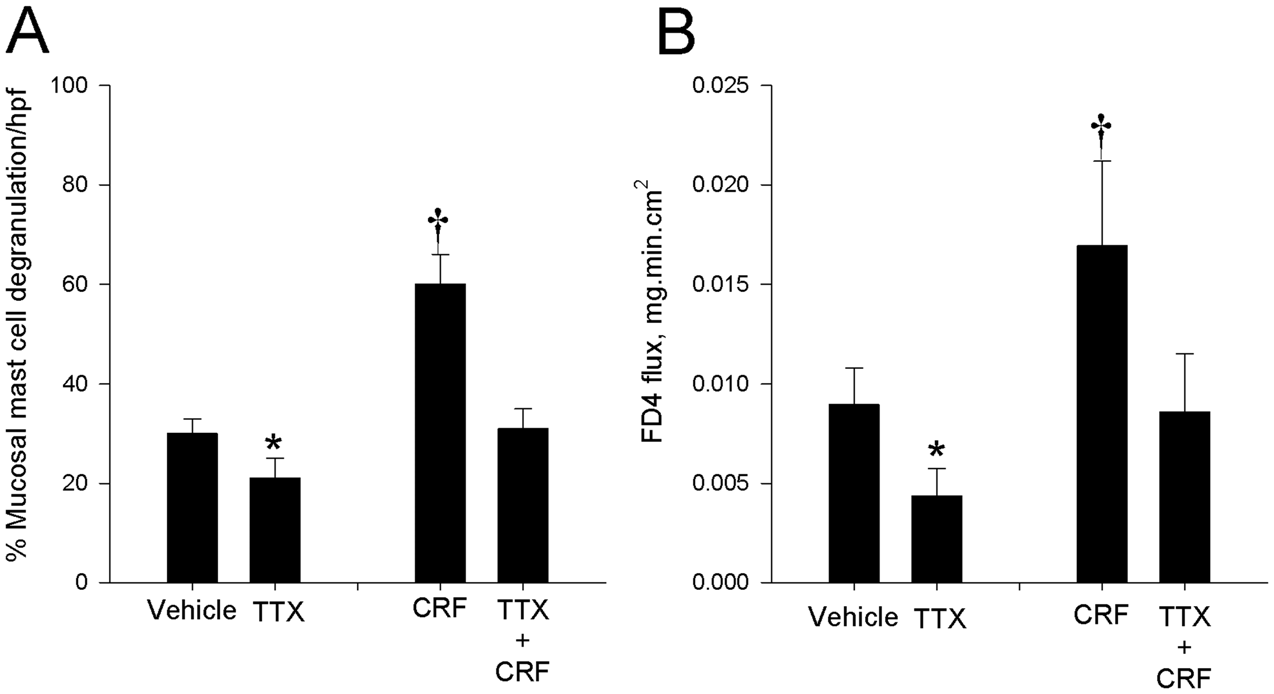
The Role of the enteric nervous system in CRF-mediated mast cell activation and FD4 flux. Toluidine blue staining of histological sections (100 X magnification) (A) and quantitation of the % of degranulated tissue mast cells (B) in porcine ileum revealed increased degranulation of mast cells in CRF–treated tissues. Treatment of ileal tissues with tetrodotoxin (TTX) inhibited mast cell degranulation and FD4 flux (C) under basal and CRF-stimulated conditions and inhibited. For histological analysis, values represent means ± SE; *n = *6 animals and are presented as the percentage of total cell count per treatment at 20× magnification. FD4 flux values represent means ± SE; *n = *6−8 animals Symbols (*,†) differs significantly by p<0.05 from vehicle control.

To determine whether or not the enteric nervous system played a critical role in CRF-mediated mast cell activation and disturbances in intestinal epithelial permeability, tissues were pretreated on Ussing chambers with the neuronal blocker, tetrodotoxin (TTX) prior to CRF exposure. TTX pretreatment blocked both baseline and CRF mediated mast cell degranulation (Figure 8A) and increases in FD4 flux (Figure 8B) thus indicating an important role of enteric neural signaling in these pathways.

## Discussion

A central pathophysiologic mechanism by which stress induces intestinal diseases, including IBS and IBD, is via disturbances in intestinal epithelial barrier function [5], [19], [20], [21], [22], [23]. These studies, utilizing an *ex vivo* Ussing chamber model with porcine ileum, demonstrate that the stress peptide, CRF, triggers disturbances in intestinal epithelial barrier function via a mechanism that involves mast cell dependent TNF-α and protease release and disruption of paracellular tight junctions. Furthermore, we show that the enteric nervous system plays a critical role in CRF-mediated mast cell activation and intestinal barrier injury.

The present studies support previous observations in rodent models in that CRF is the key mediator triggering disturbances in intestinal function including motility disorders and intestinal barrier dysfunction [6], [11], [24], [25]. Although the role of CRF in stress-induced intestinal disturbances is well-recognized, the precise mechanism by which CRF signals to trigger epithelial barrier dysfunction is not completely understood. Several groups have shown that activation of intestinal mast cells is a critical component in stress-induced intestinal barrier disturbances [11], [26], [27]. It remains unclear how CRF triggers mast cell activation and which mast cell mediators are responsible for triggering CRF mediated barrier changes. We show here that in porcine ileum, CRF induces release of mast cell proteases and TNF-α and that each of these mediators contribute significantly to CRF-induced intestinal barrier dysfunction as pharmacologic inhibition of protease activity and anti-TNF-α reduced CRF-induced elevations in intestinal permeability. The mechanisms by which mast cell proteases and TNF-α are working to induce epithelial barrier dysfunctions are currently unclear but appear to be mediating their effects on the paracellular tight junction as CRF treatment disrupted the localization of the tight junction protein occludin. It is well-known that TNF-α and proteases can trigger epithelial barrier disruption although via different mechanisms. TNF-α can potently enhance epithelial permeability through increased myosin light-chain kinase (MLCK) expression and activity [28] which alters size selectivity of the tight junction to increase paracellular flux of uncharged macromolecules [29]. In addition, increased TNF-α levels *in vivo* has recently been shown to regulate intestinal epithelial tight junctions independent of the actomyosin ring [30] by triggering caveolin-1 dependent endocytosis of occludin that could cause loss of barrier function. Additionally, TNF-α induces an increase of tight junction permeability *in vitro* through the activation of NF-κB and the subsequent down-regulation and alteration of ZO-1 protein expression [28]. MC proteases such as tryptase induce barrier dysfunction via alternate signaling mechanisms [31]. In an *in vitro* cell co-culture model using the HMC-1 human mast cell line and T84 intestinal epithelial cells, it was shown that MC derived tryptase impaired barrier function via activation proteinase-activated-receptor-2 (PAR-2) triggering tight junction breakdown and increased paracellular permeability [31]. This pathway was shown to involve Β-arrestin-dependent activation of ERK 1/2, then, regulates the reorganization of perijunctional F-actin in the tight junctions of colonocytes, causing an increase in intestinal permeability [31], [32]. Mice deficient in mast cell protease 4, a homologue for human chymase, were shown to have decreased intestinal permeability and reduced expression of claudin-3 indicating that mast cell chymase also plays an important role in regulating intestinal permeability [33]. Given that the PI cocktail used in the current study contained both tryptase and chymase inhibitors, we were not able to discern which specific mast cell proteases (tryptase and/or chymase) were responsible for CRF-mediated intestinal barrier dysfunction.

In the present study, CRF induced changes in paracellular permeability as indicated by FD4 flux; but had no effect on TER. The conflicting data between paracellular flux and TER has been observed by others [6] and may be due to activation of separate epithelial permeability pathways by CRF. For example, TNF-α has been shown to initiate MLCK dependent changes in size selectivity of the tight junction to enhance paracellular flux of uncharged macromolecules without affecting charge, while IL-13 dependent claudin-2 expression was shown to increase paracellular permeability to cation flux *in vitro* and *in vivo* without altering tight junction size selectivity [29], [30]. These ‘leak’ and ‘pore’ pathways, respectively, are quickly gaining evidence for differential regulation of paracellular permeability pathways [29], [34], and could explain the above data presenting large changes in paracellular permeability to uncharged ions but little changes in TER. Alternatively, disparate effects of CRF on FD4 flux and TER may also be due to time-dependent changes in TER. Chronic administration of CRF increases conductance and thus decreases in TER in rodents [11] whereas our *ex vivo* model would represent acute CRF effects.

Nerve-mast cell interactions have been an increasing subject of interest due to their role in several intestinal disorders [35]. The interplay between CRF, mast cells, and enteric nerves has been documented in rodents [36], [37] and humans [38]; however, the nature of these interactions remains poorly understood. In the present study, the barrier-disrupting effects of CRF were prevented by the nerve blocker, TTX demonstrating that enteric neural signaling is a critical element in CRF-mediated intestinal barrier dysfunction. This important role of the enteric nervous system in stress-induced intestinal barrier disruption has been demonstrated previously in rodent models [6], [21]; however, it remains unclear how enteric neuronal signaling modulates this important pathway. In our studies, we showed that enteric neural inhibition blocked both baseline and CRF-mediated mast cell degranulation. This suggests that enteric neural signaling may modulate intestinal barrier function under baseline and stressed conditions via the control of mast cell activation.

CRF receptors are expressed on enteric neurons [39], [40] and mast cells [41], [42] and therefore it is difficult to delineate the precise signaling pathways with the pharmacologic approach used in the present study. CRF could be mediating its effect on mast cell activation indirectly via activation of enteric neurons which release mediators such as substance P and calcitonin gene related peptide that in turn can trigger degranulation of mast cells [43], [44]. Alternatively CRF could activate mast cells via direct CRF receptor activation and this pathway could be influence by enteric neural signaling. As stated above, mast cells express CRF receptor subtypes CRF_1_ and CRF_2_ and *in vitro* studies utilizing the HMC1 mast cell line demonstrated that CRF directly stimulates the selective release of vascular endothelial growth factor (VEGF); however, tryptase and TNF-α release was not induced by CRF which in contrast to the present studies in native porcine ileal tissues [41]. The lack of effect of CRF on tryptase or TNFα observed in HMC1 cells by Cao et al. [41] is likely due to the simplified cell culture conditions and therefore lack of potentially critical co-factors that are present in native tissues. In support of this hypothesis, Asadi et al. [45] demonstrated that pre-treatment of the human mast cell line LAD2 with substance P induced CRF_1_ expression and enhanced CRF-mediated TNF-α and IL-8 release. This suggests that neural mediators may play a crucial role in priming the mast cell for CRF signaling; however, demonstration of these signaling pathways *in vivo* have yet to be demonstrated.

Overall, we show here that in porcine ileum, CRF induces the release of mast cell proteases and TNF-α which contribute significantly to CRF-induced disturbances in intestinal epithelial barrier function; furthermore these pathways are regulated by the enteric nervous system. Given the clinical significance of these pathways in stress-related intestinal disorders such as IBS and IBD, a greater understanding of how these signaling pathways are integrated during health and disease are needed.

## Materials and Methods

### Ethics Statement

The North Carolina State University Institutional Animal Care and Use Committee approved all studies.

### Animals and Intestinal Tissue Collection

Yorkshire x Hampshire cross-bred pigs of either sex and between 6−8 weeks of age were used in this study. Prior to experiments and tissue harvest, pigs were sedated with a combination of xylazine (1.5 mg im) and ketamine (11 mg/kg im) followed by euthanasia with an intravenous overdose of pentobarbital via a catheterized ear vein. Initial sedation was used to minimize stress before we obtained intestine for subsequent intestinal studies. Segments of ileum were harvested immediately after euthanasia and prepared for Ussing chamber studies.

### Chemicals and Reagents

Rat/human CRF (0.01–0.5 µM) and Astressin B (1 µM) were obtained from The Salk Institute, La Jolla, CA. CRF doses were based on comparable doses of CRF used in several experiments utilizing cell culture models [41], [45], [46] and physiological ranges found in peripheral tissues including primary rat adrenals [47] and human endometria [48]. Cromolyn sodium, porcine anti-TNF-α neutralizing antibody, protease inhibitor cocktail, c48/80, and tetrodotoxin (TTX) were obtained from Sigma Chemical. The PI cocktail contained the chymase inhibitor Soybean Trypsin Inhibitor (100 µg/mL) and the tryptase inhibitor Aprotinin (Sigma, 10 µM).

### Ussing Chamber Experiment

Ileal mucosa was stripped from the seromuscular layer in oxygenated (95% O_2_–5% CO_2_) Ringer solution (in mmol/l: 154 Na^+^, 6.3 K^+^, 137 Cl^−^, 0.3 H_2_PO_4_^-^, 1.2 Ca^2+^, 0.7 Mg^2+^, 24 HCO_3_^−^; pH 7.4). Tissues were then mounted in 1.13 cm^2^ aperture Ussing chambers, as described in previous study [5], [49]. Tissues were bathed on the serosal and mucosal sides with 10 mL of Ringer solution. The serosal bathing solution contained 10 mM glucose, which was osmotically balanced on the mucosal side with 10 mM mannitol. Bathing solutions were oxygenated (95% O_2_–5% CO_2_) and circulated in water-jacketed reservoirs maintained at 37°C. The spontaneous potential difference (PD) was measured using Ringer-agar bridges connected to calomel electrodes, and the PD was short-circuited through Ag-AgCl electrodes using a voltage clamp that corrected for fluid resistance. Tissues were maintained in the short-circuited state, except for brief intervals to record the open-circuit PD. Transepithelial electrical resistance (TER; Ω·cm^2^) was calculated from the spontaneous PD and short-circuit current (*I*_sc_), as previously described [50]. After a 30-min equilibration period on Ussing chambers, TER was recorded at 15-min intervals over a 4-hr period and then averaged to derive the basal TER values for each experimental treatment.

### Mucosal-to-serosal Fluxes of FITC-labeled Dextran

Mucosal-to-serosal fluxes of (FITC)-dextran (4 kDa; Sigma-Aldrich, St. Louis, MO) were performed at the same time as TER was measured. After a 15-min period on Ussing chambers, FITC Dextran (0.25 mM) was added to the mucosal side of Ussing chamber-mounted tissues. The FD4 was allowed to equilibrate for 15 min after which 100 µL samples (in triplicate) were taken from the serosal side of tissues at 30-minute intervals and transferred into a 96 well assay plate. The presence of FD4 fluorescence intensity of each sample was measured by fMax Fluorescence Microplate Reader (Molecular Devices, Sunnyvale, CA) and concentrations were determined from standard curves generated by serial dilution of FD4. FD4 flux was measured for 180 minutes post-CRF exposure and presented as the rate of FD4 flux (µg/min).

### Experimental Protocol

After mounting on Ussing chambers, ileal tissues were allowed to equilibrate for a 15–30 minute period until tissue TER and *I*_sc_ values stabilized. Thirty minutes prior to CRF exposure, Pharmacologic antagonists (Astressin B, cromolyn, PI, anti-TNFα, and TTX) were added to the serosal side of ileal mucosa, where the actions of these compounds are expected to exert direct effects on submucosal target cells. CRF was added to the serosal chamber and TER and FD4 flux were measured over a 180-min period.

### Histological Examination

At the end of the Ussing chamber experiments, ileal tissues fixed in 10% neutral buffered formalin, paraffin-embedded, and stained with hematoxylin and eosin for histological analysis. Tissues were visualized for changes in intestinal pathology as a result of experimental agonist treatments (Meiji Microscope Solutions, Model OMFL400).

### Mast Cell Histological Analysis Counts

Frozen sections of ileum (10 µm thick) were prepared and then fixed for 1 h in Carnoy’s fixative (60% ethanol-30% chloroform-10% glacial acetic acid). Sections were then stained for 45 min at room temperature with 0.5% toluidine blue in 0.5 N HCl in PBS. Mast cell counts were conducted on five different fields per slide from n = 4 pigs per treatment group. Data is presented as percentage of mast cells exhibiting degranulation. All histological analyses were performed by at least 2 reviewers who were blinded to experimental treatments.

### Measurement of TNF-α

Supernatant from the serosal bathing solution of Ussing Chambers was collected at time 240 min (end of experiment) for analysis of TNF-α using a commercial ELISA kit (R&D Systems) according to manufacturer’s instructions. Samples were frozen immediately upon removal from Ussing Chamber and stored at −80°C until assay.

### Tryptase Assay of Ileal Culture Supernatant

Ileal segments weighing 0.05 g were dissected into small fragments, weighed, and placed into 24 well culture plates with cRPMI +0.5% gentamicin. Tissues were incubated with CRF receptor agonist treatments for 180 minutes before supernatant was removed, centrifuged, and stored at −20°C until assay. Supernatant samples were analyzed for tryptase content using Thiobenzyl Ester Substrate assay as previously described [51].

### Immunofluorescence and Confocal Analysis

Porcine ileal sections were stained with the primary antibody as indicated by the manufacturer’s instructions. Confocal images were obtained with a 3-Laser Nikon Confocal Laser Scanning Instrument (Nikon Instruments). Images were obtained using EZ-C1 Nikon software (Silver Version 2.01). Threshold values were determined using the appropriate isotype-matched controls. A channel series approach was used to ensure no spectral overlap between fluorescent signals. Primary antibodies include occluding (mouse-anti-occludin, Invitrogen), 1∶1000. Secondary antibodies used include Fab-antibodies, Jackson Labs Inc (donkey-anti-mouse FITC), 1∶1000. Nuclei were visualized with To-Pro-3 iodide 642/661 (Invitrogen) 1∶1000.

### Statistical Analyses

Data are reported as means ± SE based on the experimental number (*n*). Data were analyzed by using a standard one-way ANOVA (Sigmastat, Jandel Scientific, San Rafael, CA). A post hoc Fisher’s least significant difference (LSD) test was used to determine differences between treatments following ANOVA. MC counts were analyzed using Mann-Whitney test, and differences considered significant if p<0.05.
